# Behavioral adaptation in diet maintains nutrient composition in an isolated, confined, and extreme environment in Antarctica

**DOI:** 10.3389/fnut.2025.1688825

**Published:** 2025-12-05

**Authors:** Bea Klos, Daniela Reinhardt, Andrea Meyer, Nadja Albertsen, Stijn Thoolen, Hannes Hagson, Carmen Possnig, Paul Enck, Isabelle Mack

**Affiliations:** 1 Internal Medicine VI, Psychosomatic Medicine and Psychotherapy, University Hospital Tübingen, Tübingen, Germany; 2 French Polar Institute Paul-Émile Victor, Brest, France; 3 Department of Clinical Medicine, Aarhus University, Aarhus, Denmark; 4 Aarhus University Hospital, Aarhus, Denmark; 5 Department of Psychiatry, Massachusetts General Hospital, Harvard Medical School, Boston, MA, United States

**Keywords:** isolated confined extreme environments, dietary intake, space mission, weight loss, nutrient intake, food frequency questionnaire

## Abstract

**Background:**

Comprehensive monitoring of dietary intake in isolated, confined, and extreme (ICE) environments is critical to elucidate physiological adaptations and to mitigate nutrition-related health risks. Although a reduction in energy intake has consistently been reported under ICE conditions, the underlying nutritional determinants remain insufficiently characterized. This study aimed to systematically examine longitudinal changes in dietary intake during a 1-year overwintering mission at Concordia Station, Antarctica.

**Methods:**

Dietary intake data were collected from 34 crewmembers across three overwintering campaigns at Concordia Station, each lasting 12 months with *ad libitum* food access. Assessments were conducted at five time points (T0: baseline; T1–T3: in-mission; T4: follow-up) using a validated Food Frequency Questionnaire covering 130 items. Nutrient intake was calculated for energy, macro- and micronutrients. Gastrointestinal symptoms were evaluated using a standardized questionnaire.

**Results:**

Participants experienced a gradual body weight loss during overwintering (−4.5% ± 6.1%), indicating a sustained energy deficit. Nevertheless, the overall macronutrient distribution remained stable over time, as opposing shifts in the intake of specific food groups balanced each other out. Although fiber intake temporarily declined (*p* < 0.001), gastrointestinal symptoms remained largely absent, suggesting that the dietary shifts did not result in notable functional impairments.

**Conclusion:**

Despite environmental constraints and limited availability of fresh foods, participants maintained a rather stable nutrient distribution, suggesting broadly adequate diet quality. The observed weight loss therefore reflects insufficient intake rather than poor diet quality. These findings highlight the adaptability of dietary behavior under ICE conditions and underscore the importance of flexible yet well-structured provisioning strategies to ensure nutritional sufficiency and physiological resilience during long-duration stays in isolated and extreme environments.

## 1 Introduction

Long-duration missions in isolated, confined, and extreme (ICE) environments place considerable demands on human physiology and behavior (1). A major challenge in extreme environments is to ensure an adequate energy intake to maintain energy balance (2–8). Recent findings from space missions (9–12) consistently associate sustained energy deficits with measurable losses in body mass (6, 7, 13–15). Findings from other ICE settings such as Antarctic stations or isolated, confined, and controlled (ICC) environments (e.g., Mars500, SIRIUS) are more heterogeneous with respect to energy intake and body weight changes (16–19). These differences likely reflect contextual variation in environment, study design, or activity levels. Nevertheless, insufficient energy intake remains a critical concern (7), given its potential to impair physiological stability and psychological resilience. Documented effects include musculoskeletal and cardiovascular decline, bone loss, immune dysfunction, and impaired mood and cognition (4, 20–27).

Beyond total energy intake, nutritional deficits often include suboptimal macronutrient distribution, which may further compromise physiological stability, psychological well-being, and operational capacity in ICE environments (4). Evidence from ICE and ICC studies indicates that carbohydrate intake generally increases, whereas fat consumption shows inconsistent patterns, with reports of both increased and decreased intake over time compared with recommended levels (4, 9–12). Protein intake, by contrast, is usually adequate or even exceeds recommendations during long-duration missions (4), although insufficient intake has been reported on shorter missions (28). To ensure adequate nutritional balance under extreme conditions, it is recommended that macronutrient intake aligns with the ranges proposed by the World Health Organization: 10%–15% of total energy from protein, 20%–35% from fat, and 45%–60% from carbohydrates (4, 7, 29). However, whether such targets are achieved or suitable in ICE settings remains unclear, given differences in activity levels, food systems, and environmental constraints. Moreover, reduced access to fresh, fiber-rich foods (30–32) may contribute to insufficient fiber intake, potentially contributing to gastrointestinal symptoms such as constipation or discomfort.

In addition to these macronutrient-related challenges, micronutrient status in ICE environments may also be affected by environmental stressors and limited dietary diversity, potentially resulting in setting-dependent deficiency patterns. In space missions, consistently reduced concentrations have been reported for vitamin K, folate, vitamin E (6), vitamin B6 (33, 34), whereas ferritin levels are often reported to be elevated. Vitamin D deficiency is well documented across ICE settings, primarily due to limited UV-B exposure, with potential consequences for calcium metabolism, bone health, and immune function (6, 16, 35). Thiamin, riboflavin, niacin, pantothenic acid, iodine, manganese, magnesium, and copper generally appear sufficient (4, 36). For most other micronutrients, evidence remains limited, underscoring the need for systematic monitoring and context-specific nutritional strategies during long-duration missions.

Despite these observations, the current evidence base on nutritional adaptations in polar ICE environments remains fragmented. Although several factors potentially affecting dietary and energy intake in ICE conditions are known (1, 6, 8, 18, 32, 37–42), the current literature is limited by small sample sizes, methodological issues, and considerable heterogeneity in research objectives. Furthermore, nutritional aspects have often received only limited attention in study designs. Terrestrial ICE environments such as Antarctic stations are essential for investigating physiological and nutritional adaptations in extreme conditions. Nutrition is a key determinant of success in long-duration ICE missions, given its role in regulating body weight and supporting physiological and psychological function (7). Developing effective countermeasures to mitigate weight loss, protect nutritional status, and address the ICE stressors requires a comprehensive understanding of the nutritional situation under extreme settings. Yet, transferability across different ICE environments must be considered with caution, as environmental conditions and food systems vary (e.g., food availability, meal preparation).

To address this research gap, we systematically assessed dietary intake, body mass changes, and gastrointestinal symptoms across multiple overwintering missions at Concordia Station in Antarctica. By combining repeated Food Frequency Questionnaires (FFQs), symptom ratings, and body weight tracking over time, we aimed to characterize nutritional constraints and physiological adaptations during prolonged stays in this setting. This approach facilitates a more detailed understanding of factors contributing to potential energy imbalances and informs the development of targeted countermeasures. Importantly, findings from this extreme setting may offer insights transferable to other ICE environments, while also being relevant in non-extreme contexts such as hospitals, care facilities, or isolated work settings (43), where appetite regulation, reduced physical activity, and limited dietary variety can also compromise adequate energy intake.

## 2 Materials and methods

### 2.1 Study design

The study followed a repeated measures design with data collection at five time periods (T0-T4): baseline/pre-departure meeting (T0) 6 weeks before departure, during the Antarctic stay at the end of summer (February, T1), mid-winter (June, T2), and end of winter (October, T3), and at follow-up 6 months after return (T4). This schedule was designed to capture long-term effects of the year-long mission at Concordia Station, including continuous isolation from February to late October/early November. All foods during the study were consumed *ad libitum*. While breakfast and between-meal snacks were individually prepared and consumed according to personal preference, lunch and dinner were freshly cooked each day by a chef and typically included a starter, main course, and dessert. At each study time point, a standardized FFQ assessed dietary intake and nutrient composition. At baseline, the questionnaire covered the previous 3 months, while at subsequent time points it referred to the period since the last measurement. Gastrointestinal symptoms were recorded in parallel using a validated symptom questionnaire. Physical activity was not assessed.

### 2.2 Study setting

Concordia Station is located on the Antarctic Plateau at Dome C, at 3,233 m (10,607 ft) above sea level. Due to low barometric pressure, this corresponds to a physiological altitude of approximately 3,800 m, resulting in chronic hypobaric hypoxia with inspired oxygen partial pressures at 21% O_2_ ranging between 132 and 136 hPa (99.01–102.01 mmHg). The environment is characterized by extreme cold, low humidity, minimal microbial exposure, sensory deprivation, and the absence of natural light during the polar night (44). Concordia Station is jointly operated by the French Polar Institute Paul-Émile Victor (IPEV) and the Italian National Antarctic Research Program (PNRA) and serves as a multidisciplinary research platform, enabling a wide range of scientific investigations in fields such as astronomy, glaciology, atmospheric sciences, and human physiology. During the Antarctic winter, the station hosts up to 16 crew members and remains completely isolated for 9 months. Meals are prepared daily by professional chefs using mostly shelf-stable, frozen, or canned foods, with limited availability of fresh fruits and vegetables. As the last resupply flight arrives in early February, the availability of fresh items such as fruits, vegetables, eggs, and dairy becomes limited within weeks, unlike at stations with year-round supply. As one of the most remote and environmentally extreme research stations on Earth, Concordia Station serves as a validated analog for spaceflight, providing valuable insights into human adaptation in ICE environments and supporting the development of countermeasures to maintain health and performance.

### 2.3 Study population

Thirty-six volunteers (81% male; mean age: 37.8 ± 11.2 years, range: 23–56 years) were recruited, comprising all crew members of the 2018/2019, 2019/2020, and 2021/2022 overwintering crews at the Antarctic Concordia research station. No data were collected during the 2020/2021 season due to COVID-19-related operational restrictions. Crew members underwent a rigorous recruitment process managed by PNRA and IPEV; the station physician was jointly selected by IPEV and the European Space Agency (ESA). This process involved medical tests, physiological and psychological assessments, and interviews, ensuring that candidates were in good physical health and had a psychological profile suitable for enduring prolonged isolation and extreme conditions. Of the 36 study participants, two were excluded due to missing baseline data, resulting in a final study population of 34 participants. Informed consent was obtained from all participants. This study was carried out in accordance with the ethical principles outlined in the Declaration of Helsinki. The study protocol was approved by the Ethics Committee of the University Hospital Tübingen, Germany (NCT03746145).

### 2.4 Data collection

Dietary intake was assessed using the validated EPIC-Norfolk Study FFQ (45). This instrument showed acceptable validity, with correlations from 0.54 (fiber) to 0.86 (alcohol) based on 24-h recalls, and 0.46 and 0.48 for protein and energy intake versus protein excretion and energy expenditure (46). The semiquantitative FFQ captures habitual dietary intake and comprises 130 predefined food and beverage items, presented either as distinct items or, in some cases, as aggregated categories (e.g., “tinned fruit”). For each item, a standard portion size is assigned. Participants were asked to indicate their average consumption frequency using a 9-point scale ranging from “never or less than once per month” to “6 or more times per day,” referring to the period between the respective measurement time points. At baseline (T0), participants were asked to report their typical intake during the previous 3 months. To improve the accuracy of fat intake estimation, additional questions were included on the type of breakfast cereals, milk, meat, and cooking fat used. Food items were grouped according to the EPIC-Norfolk food group classification, with two minor modifications to enhance physiological relevance: cocoa drinks were assigned to the “Milk and Milk Products” group due to their dairy content and nutrient profile, and calorie-free beverages were excluded from the “Beverages” group because of their negligible contribution to energy and nutrient intake. The complete list of food group assignments and portion sizes is provided in Supplementary Table 1.

To assess the plausibility of dietary reports, a validated picture-based Swiss FFQ was used as a reference (47), and food items were systematically reclassified to align with the EPIC food grouping structure. Both questionnaires were administered 1 week apart. A comparison of consumption frequency and relative portion size patterns between both FFQs was conducted for the 2019/20 and 2021/22 overwintering periods, including the 22 participants with complete data from both FFQs. Deviations from the original EPIC classification were weighted by group size and ranged from 0.76% (e.g., eggs, alcoholic beverages) to 8.33% (vegetables), indicating acceptable agreement between instruments at the item level and therefore providing a suitable reference for evaluating the plausibility of the reported intake patterns.

Potential gastrointestinal discomfort was evaluated using the Gastrointestinal Symptom Rating Scale (GSRS) (48, 49). This questionnaire consists of 15 items, each rated on a 7-point Likert scale ranging from 1 (“no discomfort at all”) to 7 (“very severe discomfort”), covering five symptom domains with moderate to good internal consistency (50): reflux (2 items, Cronbach’s α = 0.69), abdominal pain (3 items, α = 0.54), indigestion (4 items, α = 0.64), diarrhea (3 items, α = 0.76), and constipation (3 items, α = 0.73). A total score (α = 0.76) was calculated as the mean of all 15 items, with higher scores indicating greater overall symptom burden. In addition, subscale scores were computed by averaging the items within each domain, allowing for a differentiated assessment of specific gastrointestinal symptoms.

### 2.5 Statistical analysis

Statistical analyses were performed using SPSS version 28.0.0.0 (IBM Corp., Armonk, NY, USA). GraphPad Prism version 10.1.1 (GraphPad Software, San Diego, CA, USA) was used for data visualization. Descriptive statistics are reported as mean ± standard deviation for continuous variables and as percentages for categorical variables. Normality was assessed prior to analysis. Parametric data were analyzed using one-way repeated measures ANOVA with Greenhouse-Geisser correction where appropriate. Non-parametric data were analyzed using the Friedman test. Multiple testing was controlled using the false discovery rate (FDR), with Bonferroni correction applied to pairwise *post hoc* comparisons. Statistical significance was defined as *p* < 0.05 (*p* < 0.05*, *p* < 0.01**, *p* < 0.001***). To reduce potential bias associated with missing data (EPIC-FFQ: 8.3%, GSRS: 5.9%), missing data were addressed using a two-step imputation approach. Blank responses were coded as missing values and distinguished from valid “never” responses indicating non-consumption. Single-item missingness, defined as isolated unanswered items within otherwise completed questionnaires (e.g., one missing food item in the FFQ or a single symptom score in the GSRS), was resolved by mean imputation at the respective time point (2.2% of all items). Given the low overall missing rate, it was assumed that blank responses reflected accidental omissions rather than true non-consumption. For fully missing questionnaires (0.0% pre-/in-mission-questionnaires, 35.2% post-questionnaires), multiple imputation by chained equations (MICE) with fully conditional specification (Markov Chain Monte Carlo) and predictive mean matching (PMM) was applied (51, 52). Reproducibility was ensured using the Mersenne Twister algorithm with a fixed seed (2,000,000). Predictors included sex, age, baseline and post-mission body mass index (BMI), and all available baseline variables. Nominal variables from additional FFQ fat intake items (milk type, cereals, frying fat, baking fat) were imputed by mode, derived from the most frequent response across preceding time points; baseline values were used in cases of ties or high variability. As effect estimates were similar with and without imputation, results from multiple imputation are presented.

Food items from the FFQ were assigned to 14 food groups based on the modified EPIC classification. Reported frequencies were converted into monthly consumption values per item and food group. Using the standard portion sizes indicated in the FFQ, average daily intake amounts were calculated. Nutrient and energy intakes were derived using the FFQ EPIC Tool for Analysis, which translates FFQ responses into estimates of daily nutritional intake (45). To account for potential underreporting (mean energy intake based on FFQ data: 1740.1 ± 903.2 kcal at baseline, 1677.5 ± 598.2 kcal at T1, 1376.5 ± 343.8 kcal at T2, 1442.2 ± 576.9 kcal at T3, and 1332.5 ± 379.7 kcal at follow-up) and to ensure comparability (53) across participants and time periods, food group intake based on reported consumption frequencies was expressed as a proportion of the total reported intake (% of total intake). Macronutrient contributions were calculated as energy percentages (E%) using standard energy conversion factors: 4 kcal per gram for carbohydrates and protein, 9 kcal per gram for fat, and 2 kcal per gram for dietary fiber (54). Micronutrient density was defined as the amount of each micronutrient per 100 kcal, allowing energy-adjusted comparisons across time periods. Adequacy was evaluated against European Food Safety Authority (EFSA) adult reference intakes (55), adjusted for moderate to high physical activity levels (PAL 1.45–1.9), and expressed as nutrient density per 100 kcal.

## 3 Results

### 3.1 Sample characteristics

**FIGURE 1:**
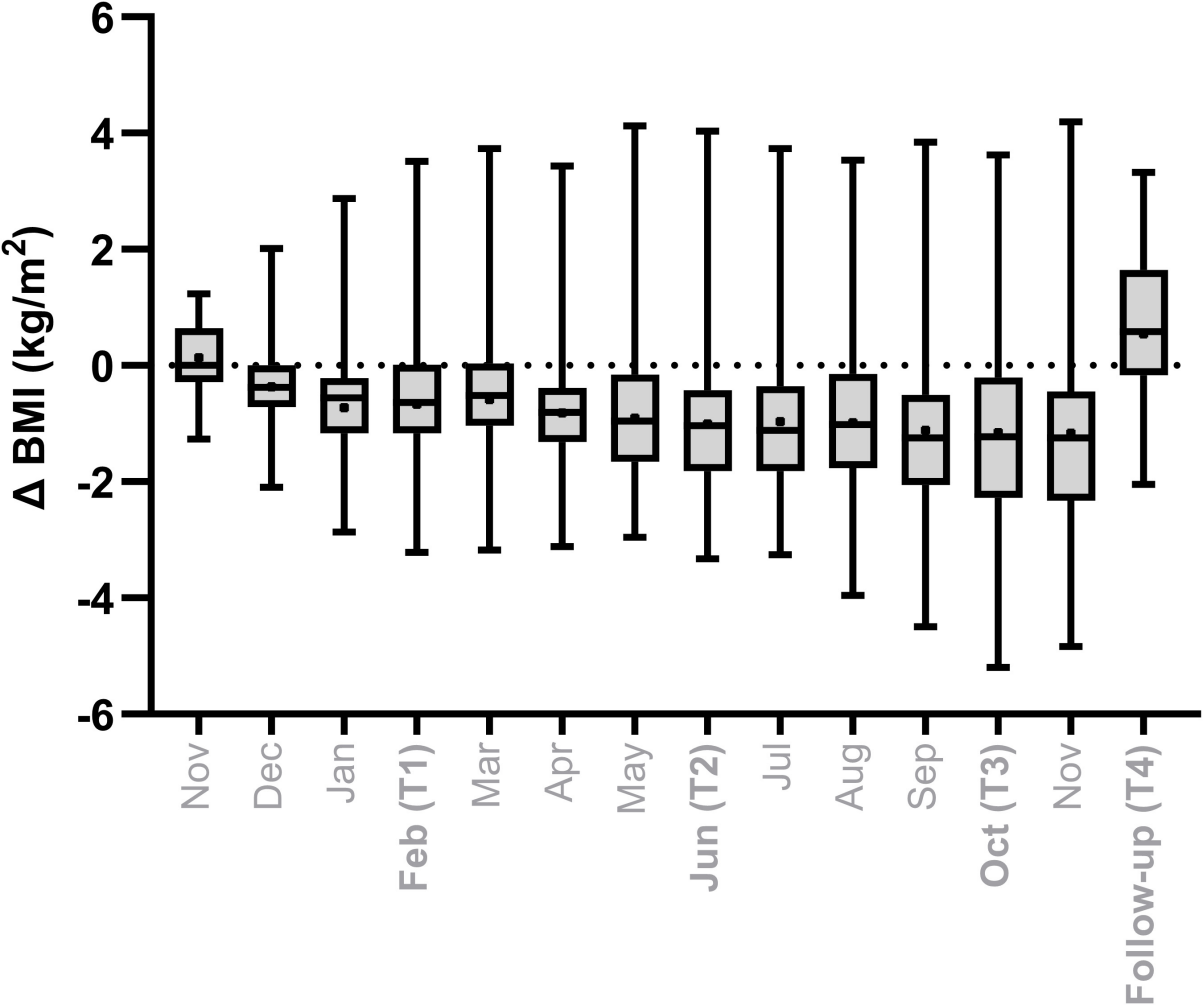
Change (Δ) in body mass index [kg/m^2^] over time relative to baseline measurement (Pre).

The analysis included 34 Caucasian participants with a mean age of 39.2 ± 10.9 years, the majority being male (79%). All participants were at a general good health. There was a subgroup of three participants who reported different forms of vegetarian diets, ranging from pescetarian to strict vegetarian. At baseline, participants had an average BMI of 25.4 ± 3.4 kg/m^2^, corresponding to a mean body weight of 76.8 ± 12.7 kg. By the end of the overwintering period, BMI had significantly decreased to 24.2 ± 3.4 kg/m^2^ (73.4 ± 13.7 kg; ANOVA, *p* = 0.016), with 47% of participants (*n* = 16) who had lost more than 5% of their baseline body weight, including three individuals with losses exceeding 10%. Conversely, one participant experienced a substantial weight gain of 16% (Figure 1).

At the 6-months follow-up, mean BMI increased to 26.0 ± 3.8 kg/m^2^ (78.5 ± 13.5 kg), surpassing baseline levels. At this point, 29% (*n* = 10) had gained more than 5% of their initial weight, including two individuals with gains >10%. Notably, three participants who had lost >5% of their body weight during overwintering remained below baseline at follow-up, with one individual continuing to lose weight post-mission.

### 3.2 Temporal shifts in food group composition during isolation with post-mission recovery toward baseline

**FIGURE 2:**
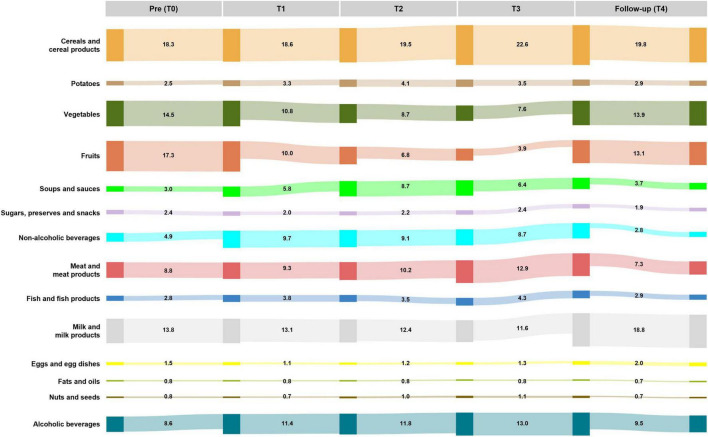
Relative changes (%) in food groups intake per day. Sankey diagram showing the share of total intake by weight contributed by each food group (% of grams) before isolation (Pre), during isolation (T1 = February, T2 = June, T3 = October), and 6 months post-isolation (Follow-up, T4). Flow widths are proportional to the percentage contribution at each time point (summing to 100% per time point).

Considerable temporal shifts in food group intake over time were consistently reflected in both absolute amounts (g/day; Supplementary Table 2) and relative intake patterns (%; Figure 2 and Supplementary Table 2). However, no significant differences in food group intake were observed between the pre- and post-mission time periods, indicating that the intake levels returned to baseline despite temporary shifts during the Antarctic stay. Given the low absolute total intake values observed during the overwintering period, subsequent analyses focus on the proportional contribution of each food group to total intake.

Cereals and cereal products consistently represented the largest share of total intake (18%–23%), without significant change over time (Friedman test, FDR = 0.321). Similarly, the intake of other carbohydrate-rich foods, such as potatoes (Friedman test, FDR = 0.181) and sugars, preserves, and snacks (Friedman test, FDR = 0.641), remained stable throughout the mission. In contrast, fruit and vegetable intake at baseline was within the recommended daily range (fruits: 2.7 ± 2.3 servings/day; vegetables: 3.8 ± 2.1 servings/day), contributing 17.3% and 14.5% to total intake, respectively, but declined significantly during the isolation phase (fruits: Friedman test, FDR < 0.001; vegetables: Friedman test, FDR < 0.001). By T3, vegetable intake had declined to around half (7.6%, 1.3 ± 1.1 servings/day), and fruit intake to less than one quarter (3.9%, 0.4 ± 0.4 servings/day) of baseline levels; both recovered by follow-up, showing a clear trend back toward pre-mission levels and daily recommendations. Conversely, carbohydrate intake as liquids increased during the Antarctic period. The intake of soups and sauces increased markedly during the isolation phase, peaking at T2 (9%) compared to pre- (3%) and post-mission levels (4%; Friedman test, FDR < 0.001). In addition, the intake of calorie-containing non-alcoholic beverages, including soft drinks and fruit juices, increased significantly during the Antarctic stay relative to both pre- and post-mission levels (Friedman test, FDR < 0.001).

Similar patterns of temporal shifts were observed for animal-based protein sources. While the share of meat and meat products increased progressively during the Antarctic stay, peaking at T3 (13%), it dropped sharply by follow-up (7%; Friedman test, FDR < 0.001). Fish and fish products also showed a moderate increase during isolation, with the highest values at T3 (4%) before returning to baseline levels (Friedman test, FDR = 0.021). In contrast, milk and milk products steadily declined throughout the mission, reaching a minimum at T3 (12%) before rising again at follow-up (19%; Friedman test, FDR = 0.001). Eggs and egg dishes followed a similar trend, with lowest proportions during the mission and a rebound post-isolation (Friedman test, FDR = 0.019).

Relative intake of fats and oils remained stable across all time points (Friedman test, FDR = 0.213), whereas nuts and seeds showed a moderate yet significant increase at T3 compared with baseline and follow-up (Friedman test, FDR = 0.002).

The relative intake of alcoholic beverages remained unchanged over time.

Reflecting the shifts in food group proportions, consumption frequencies of individual EPIC food items (Supplementary Table 3) declined over time. Nevertheless, participants continued to consume the same range of food items, indicating that overall dietary diversity was preserved despite lower intake frequencies.

### 3.3 Macronutrient composition remained stable despite changes in food group intake, with only minor and reversible declines in selected micronutrients

**FIGURE 3:**
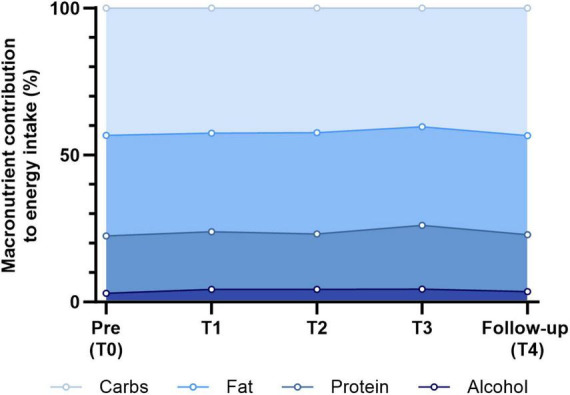
Relative changes in macronutrient composition (E%). Stacked area chart of mean energy contributions (% of total energy intake) from carbohydrates, proteins, fats, and alcohol across all time periods: carbohydrates - Pre: 43.3%, T1: 42.6%, T2: 42.4%, T3: 40.4%, follow-up: 43.4%; proteins - Pre: 19.5%, T1: 19.6%, T2: 18.9%, T3: 21.7%, follow-up: 19.3%; fats - Pre: 34.3%, T1: 33.5%, T2: 34.4%, T3: 33.6%, follow-up: 33.8%; alcohol - Pre: 3.0%, T1: 4.3%, T2: 4.3%, T3: 4.4%, follow-up: 3.5%.

Despite substantial changes in the intake of specific food groups, macronutrient composition remained stable across all time periods (Figure 3), suggesting that these shifts may have been compensatory, helping to maintain a balanced distribution of energy-yielding nutrients. Mean contributions to total energy intake ranged from 40.4 E% to 43.4 E% for carbohydrates, 18.9 E%–21.7 E% for protein, 33.5 E%–34.4 E% for fat, and 3.0 E%–4.4 E% for alcohol. No statistically significant differences were observed across time periods for any macronutrient (ANOVA, all FDR > 0.05), indicating a high degree of nutritional stability throughout the mission. The contribution of dietary fiber to total energy intake declined during the overwintering period, from 1.7 E% at baseline to 1.2 E% at T3 (Friedman test, FDR < 0.001), but returned to baseline levels by the end of the mission (Friedman test, FDR < 0.001).

Energy-adjusted consumed micronutrient density remained adequate over time for all major minerals, trace elements, and vitamins (Supplementary Table 4), except for vitamin D, which consistently was below reference values (0.14–0.16 μg/100 kcal vs. reference 0.45–0.6 μg/100 kcal, based on PAL 1.45–1.9). However, several participants reported vitamin D supplementation (*n* = 2 at Pre, *n* = 1 at T1, *n* = 7 at T2, *n* = 8 at T3, *n* = 3 at follow-up; dosage information not available), with intake frequencies ranging from once daily to less than once per month; these supplements were not included in intake calculations.

Despite statistically significant mid- and end-winter declines in the intake of calcium (Friedman test, FDR < 0.001), magnesium (Friedman test, FDR = 0.02), potassium (ANOVA, FDR < 0.001), phosphorus (Friedman test, FDR = 0.012), vitamin A (Friedman test, FDR = 0.023), and vitamin C (ANOVA, FDR < 0.001), levels recovered by follow-up. However, the micronutrient density of the diet was always good to high, and transient reductions did not fall below critical thresholds (55). Notably, no significant changes were observed across the study period for sodium and all trace elements (copper, iodine, iron, manganese, selenium, zinc), as well as for vitamins E, B1, B2, B6, B12, niacin, and folate.

### 3.4 Gastrointestinal symptoms remained low and clinically unremarkable throughout the mission

Baseline values were comparable to those reported in healthy reference populations (48). Across all time periods, mean values across all GSRS subscales remained below the threshold of 2, indicating a persistently low level of self-reported gastrointestinal symptom severity throughout the study period (Table 1).

**TABLE 1:** Gastrointestinal symptom scores (GSRS) over time.

GSRS scale	Pre	T1	T2	T3	Follow-up	*P*-value	FDR
Reflux	1.3 ± 0.4	1.3 ± 0.5	1.3 ± 0.6	1.6 ± 1.0	1.5 ± 0.6	0.268	0.537
Abdominal pain	1.5 ± 0.6	1.2 ± 0.3	1.3 ± 0.5	1.5 ± 0.8	1.4 ± 0.5	0.011*	0.064
Indigestion	1.8 ± 0.6	1.9 ± 0.7	1.9 ± 0.9	1.9 ± 0.9	1.9 ± 0.7	0.603	0.673
Diarrhea	1.5 ± 0.7	1.7 ± 0.7	1.7 ± 0.9	1.8 ± 1.1	1.7 ± 0.8	0.145	0.435
Constipation	1.4 ± 0.5	1.3 ± 0.4	1.3 ± 0.5	1.3 ± 0.5	1.4 ± 0.3	0.673	0.673
Total score	1.5 ± 0.4	1.5 ± 0.4	1.5 ± 0.6	1.6 ± 0.7	1.6 ± 0.4	0.504	0.673

Mean (±SD) total and subscale scores of the Gastrointestinal Symptom Rating Scale (GSRS) before isolation (Pre), during isolation (T1 = February, T2 = June, T3 = October), and 6 months post-isolation (Follow-up, T4). Scores range from 1 to 7, with higher values indicating greater symptom severity. Statistical significance was assessed using repeated measures analysis with false discovery rate (FDR) correction. Pairwise comparisons between time periods were Bonferroni-adjusted. Statistical significance was defined as *p* < 0.05, with significant effects indicated by an asterisk (*).

Notably, abdominal pain scores were significantly lower at the beginning of the mission compared to baseline (Friedman test, *p* = 0.011), but appeared to return to baseline levels thereafter. No other subscale showed statistically significant changes over time, a pattern also reflected in the stability of the total GSRS score. Median total scores remained between 1.4 and 1.6 [IQR 1.1–2.0], further indicating a lack of clinically relevant gastrointestinal complaints throughout the study.

## 4 Discussion

This study investigated dietary intake and gastrointestinal tolerance during prolonged exposure to an ICE environment. Unintended weight loss in such settings raises concerns about energy balance and nutritional adaptation, particularly when it may compromise health or performance.

A key physiological finding of this study was the consistent reduction in body weight over the course of the overwintering period, despite *ad libitum* food availability, suggesting a persistent negative energy balance [≈75 kcal/day, estimated using Wishnofsky’s rule (56)]. Considerable inter-individual variability in weight change was observed, which may reflect underlying mechanisms related to baseline body composition, physical activity, or coping strategies; however, underlying potential mechanisms remain unclear. The overall pattern of progressive weight loss parallels findings from space missions and points to shared physiological stressors such as chronic hypoxia (17, 18), sensory impairments (30), or circadian misalignment (57) due to constant artificial lighting, reduced physical activity, and psychological strain. However, important differences in dietary systems (e.g., availability of a kitchen and frozen foods) and environmental factors [e.g., microgravity (58, 59)] limit the direct transferability of findings to spaceflight contexts. Nevertheless, the convergence of relevant physiological stressors at Concordia Station could provide a useful approach to study countermeasures against weight and muscle loss under ICE conditions.

A detailed understanding of behavioral and physiological determinants of energy balance in ICE environments is essential for designing targeted nutritional and operational strategies to mitigate unintended weight loss and preserve lean body mass, particularly when such changes may compromise health or performance (6, 7, 13–15, 58, 59). Irregular eating patterns, such as unstructured breakfasts and skipped snacks, may have played a role in the observed energy deficit. At Concordia Station, only lunch and dinner were offered as structured, communal meals, while breakfast and between-meal snacks were typically self-organized. Given the relative dietary flexibility in this ICE setting compared to e.g., space missions (28, 60) or ICC settings (61–64), implementing more structured meal plans may help support energy balance and reduce the risk of unintended weight loss. In addition to structural modifications, physiological mechanisms of appetite regulation may offer further potential to support adequate energy intake (65). Gastric distension constitutes a primary satiety signal (66, 67) and may be particularly relevant under ICE conditions, where external modulators such as food variety, olfactory stimuli, and social context are diminished. As satiety is largely volume-driven (66, 67), increasing portion size is of limited feasibility in such settings. Instead, enhancing the energy density of meals by increasing caloric content without expanding volume may facilitate sufficient energy intake (68). Adjusting the composition of familiar meals in this direction could therefore represent a practical and physiologically plausible approach to support intake under such conditions. However, as this strategy often relies on increasing fat content, its applicability remains unclear and should be carefully tested in future studies (4) before deriving general recommendations. It should be noted, however, that the contribution of these behavioral and physiological determinants to energy imbalance and thus weight loss remains uncertain, and additional factors such as reduced food acceptability may also have played a role, which should be further considered in future research. Furthermore, FFQ data consistently indicated implausibly low intake, confirmed by cross-validation (data not shown). Reported intake remained unrealistically low despite weight regain, indicating habitual misreporting (69–72), possibly influenced by temporary chemosensory impairments (73), as frequently reported during the mission (74). Additionally, weight changes may bias intake perception (75), especially under psychological strain.

Notably, the relative contributions of carbohydrates, protein, fat, and alcohol to total energy remained stable throughout the mission, suggesting that macronutrient distribution was not a primary driver of the observed energy imbalance but rather indicative of general underconsumption. To our knowledge, such consistency has rarely been reported in Antarctic settings, and available data are often limited by insufficient detail on dietary intake and assessment methods (76). Some earlier Antarctic studies reported shifts toward higher carbohydrate and lower fat intake, often accompanied by weight gain (16, 19), yet these findings were largely based on brief FFQs with few measurement points and limited methodological transparency. The accuracy of intake estimates and the extent of underreporting remain unclear. Simpson (19) highlighted these shortcomings, noting that most included investigations were older, conducted under different environmental conditions, and relied on less advanced dietary assessment tools. Importantly, all were carried out at coastal or sea-level stations without hypobaric hypoxia, which limits comparability to high-altitude inland stations such as Concordia. Similar macronutrient shifts have also been described in spaceflight, often attributed to microgravity-related appetite suppression (11) or diet monotony (10). In contrast, the stable macronutrient intake at Concordia may reflect compensatory adjustments to environmental conditions, including unrestricted food access, consistent variety, and higher physical activity levels compared with spaceflight. This pattern suggests a degree of regulatory flexibility that may help maintain nutrient balance despite environmental constraints.

While overall macronutrient distribution remained stable, individual food group contributions varied considerably, whereas dietary diversity was maintained. During the overwintering period, opposing trends were observed within major macronutrient-based food groups: among protein-contributing food groups, a marked reduction in milk and milk products and eggs and egg dishes intake contrasted with a significant rise in meat and meat products. Similarly, while intakes of fruits and vegetables declined, consumption of soups and sauces and non-alcoholic beverages increased, indicating a shift toward liquid carbohydrate sources without altering the overall carbohydrate contribution to total intake. As both reduced and increased groups were low in energy density, these substitutions likely did not affect overall energy density of the diet. Nevertheless, the persistent weight loss suggests insufficient energy intake in some crew members, underscoring the need to optimize both meal composition and implementation under ICE conditions.

In parallel, these shifts reduced fiber intake, likely reflecting the limited availability of fresh, fiber-rich foods. Although reduced fiber is generally considered a risk factor for gastrointestinal problems, no increase in complaints was observed, indicating that the Concordia diet was functionally well tolerated despite reduced fiber. This contrasts with spaceflight studies reporting frequent gastrointestinal issues during extended missions (77). The absence of objective markers, however, limits conclusions on long-term effects. Enhancing diets with fiber-rich foods appears to be a sensible strategy to improve gastrointestinal health and overall nutrient adequacy (78), but its feasibility and effectiveness under ICE conditions require further investigation. When carefully balanced, such adaptations could help restore fiber to physiologically beneficial levels while also promoting a more sustainable plant-to-animal ratio aligned with planetary health goals.

Despite shifts in food group intake, overall micronutrient density consistently met recommendations for moderate to high physical activity [PAL 1.45–1.9 (55)], indicating that the food system was largely adequate under mission constraints. Vitamin D was the only exception, with intake remaining substantially below reference values at all time points. Given the limited dietary sources insufficient to meet vitamin D requirements (e.g., eggs, fish, milk, meat), such a gap was expected and aligns with previous Antarctic and spaceflight studies reporting insufficient vitamin D supply and the lack of endogenous synthesis under polar conditions (16, 35, 79, 80). Although status could not be measured directly, the persistently low intake strongly suggests inadequate supply. Evidence from earlier studies shows that proactive screening (81) and targeted supplementation can effectively reduce deficiency risk (35), supporting the recommendation that vitamin D supplementation should be considered a standard preventive measure in ICE missions.

### 4.1 Strengths and limitations

This study provides a rare, year-long dataset from Concordia Station, collected under standardized ICE conditions across five time periods, including baseline and follow-up. The sample size (*n* = 34) exceeds that of most ICE studies (median *n* = 4–10) (82, 83), enabling more robust longitudinal analyses. A particular strength lies in the dietary assessment: the use of a second independently administered FFQ improved internal consistency by cross-validating reported intake frequencies and portion sizes. Moreover, by analyzing food-group contributions as proportions of total energy and nutrients, rather than relying on absolute intakes (53), we reduced the impact of individual misreporting and provided a comprehensive 1-year dietary assessment under standardized ICE conditions.

Nonetheless, energy balance estimates remain based on self-reported data and are therefore subject to the inherent limitations of FFQs (46, 75). The lack of objective intake and expenditure measures, such as weighed food records or doubly labeled water, further limits the precision of energy balance quantification. Similarly, without biochemical analyses, baseline micronutrient status remains unknown, precluding conclusions on the adequacy of nutrient supply. Furthermore, this work is limited to energy imbalance caused by dietary shifts; other processes such as cold-induced thermogenesis or stress-related hormonal changes may also have contributed, but their impact was not assessed and is therefore not further considered here. Importantly, a relatively high level of missing data at follow-up, largely due to personal reasons (e.g., work or travel), may have influenced post-mission results and should be considered when interpreting outcomes. Finally, variability in environmental, logistical, nutritional, and physiological conditions across ICE environments may constrain the transferability of the findings to other ICE settings.

## 5 Conclusion

Despite the gradual weight loss observed during the mission, nutritional intake at Concordia Station was adequate in terms of macronutrient distribution and micronutrient density. Insufficient energy intake was likely influenced by environmental conditions and unstructured eating patterns rather than by limited food availability, although other contributors, such as the acceptability of certain food items or groups, cannot be excluded. While causality for the observed weight loss remains uncertain, structured meal timing and increased energy density across meals are modifiable nutritional factors that could potentially be targeted in future missions. Maintaining dietary adequacy during prolonged ICE missions requires strategies to enhance overall dietary variety by improving food availability, ensuring storage stability, and providing flexible alternatives when fresh options are limited, such as nutrient-rich preserved or liquid components. Although overall nutrient supply remained adequate, with sufficient macro- and micronutrient intake and no gastrointestinal complaints, the limited access to fresh fruits, vegetables, and fiber-rich foods may still warrant consideration in future menu planning for prolonged isolation. Moreover, the potential limitation in dietary vitamin D supply, in combination with the absence of sunlight necessary for endogenous synthesis, makes systematic supplementation essential during Antarctic overwintering. However, generalizability across ICE environments remains limited; thus, the findings should be interpreted with caution and within the environmental and operational context of Concordia Station.

## Data Availability

The raw data supporting the conclusions of this article will be made available by the authors, without undue reservation.
