# Supplementary material for: Behavioral adaptation in diet maintains nutrient composition in an isolated, confined, and extreme environment in Antarctica
Source: Front Nutr. 2025 Dec 5;10:1688825. doi: 10.3389/fnut.2025.1688825 (PMC12715607; doi:10.3389/fnut.2025.1688825)
Supplement: Supplementary file 1 [file Data_Sheet_1.pdf]

Behavioral Adaptation in Diet Maintains Nutrient Composition in an Isolated, Confined, and Extreme Environment in Antarctica

Supplementary Material

1 Supplementary Tables

Supplementary Table S1: Classification of EPIC food items into food groups (partially modified from the original EPIC categorization).

**Meat and Meat products**	Beef; Beef burgers; Pork; Lamb; Chicken or turkey; Bacon; Ham; Corned beef; Sausages; Savory pies; Liver; Lasagna, moussaka
**Fish and Fish products**	Fish in batter (as in fish and chips); Fish fingers and fish cakes; Other white fish; Oily fish; Shellfish; Fish roe
**Cereals and Cereal products**	White bread and rolls; Brown bread and rolls*; Whole meal bread and rolls; Cream crackers; Crispbread; Porridge, Readybrek; Breakfast cereals; White rice; Brown rice; White or green pasta; Whole meal pasta; Pizza; Sweet biscuits, chocolate; Sweet biscuits, plain; Home baked cakes; Readymade cakes; Home baked buns; Readymade buns; Home baked fruit pies; Readymade fruit pies; Home baked sponge puddings; Readymade sponge puddings; Milk puddings
**Potatoes**	Boiled, mashed, instant or jacket potatoes; Chips; Roast potatoes; Potato salad
**Milk and milk products**	Single or sour cream; Double or clotted cream; Low fat yoghurt, fromage frais; Full fat or Greek yoghurt; Dairy desserts; Cheese, e.g. Cheddar, Brie, Edam; Cottage cheese, low fat soft cheese; Coffee whitener; Cocoa, hot chocolate; Horlicks, Ovaltine
**Egg and egg dishes**	Eggs as boiled, fried, scrambled, etc.; Quiche
**Soups and sauces**	Low-calorie salad cream; Salad cream; French dressing; Other salad dressing*; Vegetable soups; Meat soups; Sauces; Tomato ketchup; Pickles, chutney; Marmite, Bovril
**Fats and oils**	Butter; Hard margarine; Polyunsaturated margarine; Other soft margarine; Low fat spread; Very low-fat spread
**Sugars; preserves and snacks**	Ice cream, choc ices; Chocolates, single or squares; Chocolate snack bars, Crunchie; Sweets, toffees, mints; Sugar added to tea, coffee, cereal; Crisps or other packet snacks; Jam, marmalade, honey

**Nuts and seeds**	Peanuts or other nuts; Peanut butter
**Non-alcoholic beverages**	Fizzy soft drinks, e.g. Coca Cola, lemonade; Pure fruit juice (100%); Fruit squash or cordial
**Alcoholic beverages**	Wine; Beer, lager or cider; Port, sherry, vermouth, liqueurs; Spirits, e.g. gin, brandy, whisky, vodka
**Fruits**	Apples; Pears; Oranges, satsumas, mandarins; Grapefruit; Bananas; Grapes; Melon; Peaches, plums, apricots; Strawberries, raspberries, kiwi fruit; Tinned fruit; Dried fruit; Avocado
**Vegetables**	Carrots; Spinach; Broccoli, spring greens, kale; Brussels sprouts; Cabbage; Peas; Green beans, broad beans, runner beans; Marrow, courgette; Cauliflower; Parsnips, turnips, swedes; Leeks; Onions; Garlic; Mushrooms; Sweet peppers; Beansprouts; Green salad, lettuce, cucumber, celery; Watercress; Tomatoes; Sweetcorn; Beetroot; Coleslaw; Baked beans; Dried lentils, beans, peas*; Tofu or soya meat

Notes: *Due to their absence in certain language versions of the food frequency questionnaire, these items were reported infrequently and were therefore excluded from the majority of analyses.

Supplementary Table S2: Daily intake of the EPIC food groups.

Food group	Pre	T1	T2	T3	Follow-up	FDR	Pairwise comparison
Cereal and cereal products [g/d]	263.7 ± 161.2	256.3 ± 110.5	231.2 ± 93.9	230.8 ± 119.5	229.9 ± 125.8	0.278	n.s.
Cereal and cereal products [%]	18.3 ± 7.1	18.6 ± 7.6	19.5 ± 8.0	22.6 ± 9.9	19.8 ± 10.3	0.321	n.s.
Potatoes [g/d]	36.5 ± 27.2	44.5 ± 29.7	49.4 ± 40.1	35.2 ± 26.7	34.2 ± 18.6	**0.028***	n.s.
Potatoes [%]	2.5 ± 1.9	3.3 ± 2.2	4.1 ± 3.2	3.5 ± 2.3	2.9 ± 1.3	0.181	n.s.
Vegetables [g/d]	198.6 ± 117.4	153.8 ± 103.4	105.0 ± 75.7	78.4 ± 70.5	166.4 ± 92.2	**<0.001*** **	**B, C , E, F, I , K**
Vegetables [%]	14.5 ± 7.1	10.8 ± 7.0	8.7 ± 6.2	7.6 ± 6.1	13.9 ± 6.2	**<0.001*** **	**A, B, C, F, I, K**
Fruits [g/d]	238.5 ± 184.9	143.3 ± 142.5	81.8 ± 89.3	41.1 ± 45.6	153.8 ± 97.9	**<0.001*** **	**A, B, C, F, I, K**
Fruits [%]	17.3 ± 10.0	10.0 ± 10.2	6.8 ± 7.5	3.9 ± 3.9	13.1 ± 7.5	**<0.001*** **	**A, B, C, I, K**
Soups and sauces [g/d]	43.7 ± 33.2	84.9 ± 47.4	104.8 ± 75.3	64.6 ± 56.7	43.7 ± 36.0	**<0.001*** **	**A, B, F, G, H, I**
Soups and sauces [%]	3.0 ± 2.3	5.8 ± 2.6	8.7 ± 6.2	6.4 ± 5.6	3.7 ± 2.7	**<0.001*** **	**A, B, I**
Sugars, preserves and snacks [g/d]	36.2 ± 28.5	29.4 ± 23.6	25.4 ± 17.6	24.9 ± 20.4	22.8 ± 18.6	0.098	n.s.
Sugars, preserves and snacks [%]	2.4 ± 1.6	2.0 ± 1.3	2.2 ± 1.4	2.4 ± 1.6	1.9 ± 1.6	0.641	n.s.
Non-alcoholic beverages [g/d]	78.1 ± 99.5	158.6 ± 178.6	131.2 ± 183.0	107.9 ± 122.3	34.4 ± 42.2	**<0.001*** **	**G, I, K**
Non-alcoholic beverages [%]	4.9 ± 5.3	9.7 ± 8.7	9.1 ± 10.1	8.7 ± 8.1	2.8 ± 3.1	**<0.001*** **	**G, I, K**
Meat and meat products [g/d]	120.1 ± 68.0	135.5 ± 79.0	116.5 ± 53.7	143.8 ± 186.6	83.2 ± 53.4	0.166	n.s.
Meat and meat products [%]	8.8 ± 5.5	9.3 ± 4.3	10.2 ± 5.3	12.9 ± 8.9	7.3 ± 4.7	**<0.001*** **	**K**
Fish and fish products [g/d]	38.9 ± 23.4	54.9 ± 41.0	40.1 ± 32.7	42.2 ± 27.9	33.8 ± 22.8	**0.008** **	**A, E, G**
Fish and fish products [%]	2.8 ± 1.7	3.8 ± 2.7	3.5 ± 3.0	4.3 ± 3.1	2.9 ± 1.7	**0.033***	n.s.
Milk and milk products [g/d]	223.2 ± 197.8	183.7 ± 148.3	159.9 ± 172.4	135.2 ± 159.3	229.7 ± 191.1	**0.001** **	**C, K**
Milk and milk products [%]	13.8 ± 9.3	13.1 ± 10.9	12.4 ± 13.3	11.6 ± 12.6	18.8 ± 12.9	**0.002** **	**C, I, K**
Eggs and egg dishes [g/d]	20.7 ± 22.8	14.3 ± 12.8	14.6 ± 18.4	14.1 ± 21.9	22.7 ± 23.4	**0.002** **	**C, K**
Eggs and egg dishes [%]	1.5 ± 1.8	1.1 ± 1.0	1.2 ± 1.3	1.3 ± 1.7	2.0 ± 1.8	**0.036***	**G, K**
Fats and oils [g/d]	11.0 ± 13.3	10.8 ± 9.7	9.0 ± 7.9	8.1 ± 9.2	7.7 ± 10.2	**0.048***	**G**
Fats and oils [%]	0.8 ± 0.9	0.8 ± 0.7	0.8 ± 0.7	0.8 ± 0.8	0.7 ± 0.8	0.249	n.s.
Nuts and seeds [g/d]	8.6 ± 14.4	8.7 ± 9.3	12.6 ± 14.8	11.8 ± 14.7	9.2 ± 23.7	**0.004** **	**B, I**
Nuts and seeds [%]	0.8 ± 1.7	0.7 ± 0.9	1.0 ± 1.3	1.1 ± 1.3	0.7 ± 1.7	**0.005** **	**I, K**
Alcoholic beverages [g/d]	113.6 ± 95.5	163.7 ± 196.1	146.8 ± 167.4	135.3 ± 157.6	111.5 ± 88.4	0.635	n.s.
Alcoholic beverages [%]	8.6 ± 7.4	11.4 ± 10.8	11.8 ± 9.4	13.0 ± 12.3	9.5 ± 7.0	0.101	n.s.

Notes: Daily intake [g] and relative daily intake [%] of food groups presented as mean ± SD. Statistical significance was assessed using repeated measures analysis with false discovery rate (FDR) correction. Significance levels: *FDR = .01–.05; **FDR = .001–.01; ***FDR < .001. Pairwise comparisons from the Bonferroni-adjusted post hoc test are denoted by letters: A = Pre–T1, B = Pre–T2, C = Pre–T3, D = Pre– Follow-up, E = T1–T2, F = T1–T3, G = T1– Follow-up, H = T2–T3, I = T2– Follow-up, K= T3– Follow-up. *Abbreviations:* n.s. = not significant.

Supplementary Table S3: Descriptive and statistical analysis of individual food items grouped by food group.

Meat and meat products: Frequency of consumption (Median [P25-P75])

	Pre	T1	T2	T3	Follow-up	FDR	Pairwise comparison
Beef	**4** [2 - 12]	**12** [4 - 12]	**4** [4 - 12]	**4** [4 - 12]	**4** [2 - 12]	**0.029***	**G**
Burger	**2** [0 - 2]	**2** 0 - 2]	**2** [0 - 2]	**2** [0 - 2]	**2** [0 - 2]	0.600	n.s.
Pork	**2** [2 - 12]	**4** [2 - 12]	**4** [2 - 12]	**4** [2 - 4]	**2** [0 - 4]	0.051	n.s.
Lamb	**0** [0 - 2]	**2** [0 - 4]	**4** [2 - 4]	**2** [2 - 3]	**0** [0 - 2]	**< .001*****	**A, B, G, I**
Chicken	**4** [2 - 12]	**4** [2 - 12]	**4** [4 - 6]	**4** [2 - 12]	**4** [2 - 12]	0.815	n.s.
Bacon	**0** [0 - 2]	**0** [0 - 2]	**0** [0 - 2]	**0** [0 - 2]	**0** [0 - 2]	0.231	n.s.
Ham	**4** [2 - 12]	**2** [0 - 4]	**2** [2 - 4]	**2** [0 - 3]	**2** [2 - 4]	**0.021***	**C**
Corned Beef	**0** [0 - 2]	**0** [0 - 4]	**0** [0 - 2]	**0** [0 - 2]	**0** [0 - 2]	0.373	n.s.
Sausages	**2** [0 - 2]	**2** [0 - 2]	**2** [0 - 2]	**1** [0 - 2]	**2** [0 - 2]	0.631	n.s.
Savoury Pies	**0** [0 - 0]	**0** [0 - 0]	**0** [0 - 0]	**0** [0 - 0]	**0** [0 - 0]	0.267	n.s.
Liver	**0** [0 - 0]	**0** [0 - 0]	**0** [0 - 0]	**0** [0 - 0]	**0** [0 - 0]	0.613	n.s.
Lasagne	**0** [0 - 2]	**2** [1.5 - 2]	**2** [0 - 2]	**2** [0 - 2]	**0** [0 - 2]	**< .001*****	**G, I**

Fish and fish products: Frequency of consumption (Median [P25-P75])

	Pre	T1	T2	T3	Follow-up	FDR	Pairwise comparison
Fried Fish	**0** [0 - 2]	**2** [0 - 2]	**2** [0 - 2]	**0** [0 - 2]	**0** [0 - 2]	0.060	n.s.
Fish Fingers	**0** [0 - 0]	**0** [0 - 2]	**0** [0 - 1]	**0** [0 - 1]	**0** [0 - 2]	0.651	n.s.
White Fish	**4** [2 - 4]	**4** [2 - 12]	**2** [2 - 4]	**4** [2 - 6]	**2** [2 - 4]	**0.047***	n.s.
Oily Fish	**3** [2 - 4]	**2** [2 - 4]	**2** [0 - 3]	**2** [0 - 4]	**2** [2 - 4]	0.174	n.s.
Shellfish	**2** [0 - 2]	**2** [0 - 2]	**2** [0 - 2]	**2** [0 - 2]	**2** [0 - 2]	0.882	n.s.
Roe	**0** [0 - 0]	**0** [0 - 0]	**0** [0 - 0]	**0** [0 - 0]	**0** [0 - 0]	0.247	n.s.

Cereal and cereal products: Frequency of consumption (Median [P25-P75])

	Pre	T1	T2	T3	Follow-up	FDR	Pairwise comparison
White Bread	**12** [2 - 28]	**22** [12 - 28]	**12** [4 - 28]	**25** [12 - 70]	**12** [12 - 22]	**0.016***	**C**
Wholemeal Bread	**4** [2 - 22]	**8** [0 - 15]	**3** [0 - 15]	**0** [0 - 2]	**4** [2 - 12]	**0.002****	**C, F**
Crackers	**0** [0 - 2]	**0** [0 - 0.5]	**0** [0 - 0]	**0** [0 - 0]	**1** [0 - 2]	**0.001****	n.s.
Crispbread	**0** [0 - 0]	**0** [0 - 0]	**0** [0 - 0]	**0** [0 - 0]	**0** [0 - 1]	**0.003****	n.s.
Porridge	**0** [0 - 0]	**0** [0 - 0]	**0** [0 - 0]	**0** [0 - 0]	**0** [0 - 0]	0.182	n.s.
Cereal	**0** [0 - 6]	**0** [0 - 22]	**0** [0 - 22]	**0** [0 - 5]	**2** [0 - 4]	0.631	n.s.
White Rice	**4** [2 - 12]	**4** [2 - 4]	**4** [2 - 12]	**4** [2 - 12]	**2** [2 - 4]	**0.048***	n.s.
Brown Rice	**0** [0 - 2]	**0** [0 - 0]	**0** [0 - 0]	**0** [0 - 0]	**1** [0 - 2]	**< .001*****	n.s.
White Pasta	**12** [4 - 22]	**12** [12 - 22]	**12** [10 - 22]	**12** [12 - 22]	**12** [4 - 15]	0.067	n.s.
Wholemeal Pasta	**0** [0 - 2]	**0** [0 - 0]	**0** [0 - 0]	**0** [0 - 0]	**2** [0 - 4]	**< .001*****	**K**
Pizza	**2** [2 - 4]	**4** [4 - 4]	**2** [2 - 4]	**2** [2 - 4]	**4** [2 - 4]	**0.028***	n.s.
Chocolate Biscuit	**4** [2 - 22]	**12** [4 - 24]	**8** [2 - 22]	**4** [0 - 28]	**4** [2 - 12]	0.158	n.s.
Plain Biscuit	**2** [0 - 12]	**3** [0 - 15]	**1** [0 - 12]	**3** [0 - 12]	**2** [0 - 4]	0.525	n.s.
Homebaked Cake	**0** [0 - 2]	**2** [0 - 4]	**2** [0 - 3]	**0** [0 - 2]	**0** [0 - 2]	**0.029***	n.s.
Readymade Cake	**0** [0 - 2]	**0** [0 - 2]	**0** [0 - 2]	**0** [0 - 2]	**0** [0 - 2]	0.768	n.s.
Homebaked Buns	**0** [0 - 2]	**2** [0 - 4]	**1** [0 - 2]	**0** [0 - 2]	**2** [0 - 2]	0.216	n.s.
Readymade Buns	**2** [2 - 4]	**2** [0 - 3]	**0** [0 - 2]	**0** [0 - 0]	**2** [0 - 4]	**< .001*****	**B, C, K**
Homebaked Fruit Pies	**1** [0 - 2]	**2** [0 - 4]	**2** [0 - 4]	**0** [0 - 4]	**1** [0 - 2]	**0.006****	n.s.

	Pre		T1		T2		T3		Follow-up		FDR	Pairwise comparison
Readymade Fruit Pies	**0**	[0 - 0]	**0**	[0 - 2]	**0**	[0 - 0]	**0**	[0 - 0]	**0**	[0 - 0]	0.080	n.s.
Homebaked Sponge	**0**	[0 - 0]	**0**	[0 - 0]	**0**	[0 - 0]	**0**	[0 - 0]	**0**	[0 - 2]	0.051	n.s.
Readymade Sponge	**0**	[0 - 0]	**0**	[0 - 0]	**0**	[0 - 0]	**0**	[0 - 0]	**0**	[0 - 0]	**0.009****	n.s.
Milk Puddings	**0**	[0 - 2]	**0**	[0 - 0]	**0**	[0 - 2]	**0**	[0 - 0]	**0**	[0 - 2]	**0.012***	n.s.

Potatoes: Frequency of consumption (Median [P25-P75])

	**Pre**		**T1**		**T2**		**T3**		**Follow-up**		**FDR**	**Pairwise comparison**
Boiled Potatoes	**2**	[0 - 4]	**2**	[2 - 4]	**2**	[2 - 4]	**2**	[0 - 2]	**2**	[0 - 2]	0.077	n.s.
Chips	**2**	[0 - 4]	**2**	[2 - 4]	**2**	[2 - 4]	**2**	[2 - 4]	**2**	[2 - 4]	0.154	n.s.
Roast Potatoes	**2**	[0 - 4]	**2**	[2 - 4]	**2**	[2 - 4]	**2**	[0 - 3]	**2**	[2 - 4]	**0.021***	n.s.
Potato Salad	**0**	[0 - 2]	**0**	[0 - 2]	**0**	[0 - 0]	**0**	[0 - 0]	**0**	[0 - 2]	**0.006****	n.s.

Milk and milk products: Frequency of consumption (Median [P25-P75])

	**Pre**		**T1**		**T2**		**T3**		**Follow-up**		**FDR**	**Pairwise comparison**
Milk	**28**	[0 - 28]	**28**	[0 - 28]	**0**	[0 - 28]	**0**	[0 - 28]	**28**	[0 - 28]	**0.01****	n.s.
Single Cream	**0**	[0 - 4]	**2**	[0 - 4]	**1**	[0 - 4]	**0**	[0 - 4]	**2**	[0 - 4]	0.769	n.s.
Double Cream	**0**	[0 - 4]	**0**	[0 - 2.5]	**1**	[0 - 2]	**0**	[0 - 2]	**0**	[0 - 2]	0.597	n.s.
Lowfat Yogurt	**2**	[0 - 12]	**2**	[0 - 4]	**0**	[0 - 2]	**0**	[0 - 12]	**2**	[0 - 4]	**0.003****	**B**
Fullfat Yogurt	**2**	[0 - 2]	**0**	[0 - 2]	**0**	[0 - 2]	**0**	[0 - 0]	**2**	[0 - 2]	**0.005****	n.s.
Dairy Dessert	**2**	[0 - 4]	**2**	[0 - 4]	**0**	[0 - 2]	**0**	[0 - 0]	**0**	[0 - 2]	**< .001*****	**C**
Cheese	**4**	[0 - 12]	**4**	[0 - 12]	**3**	[2 - 12]	**4**	[2 - 12]	**4**	[2 - 12]	0.461	n.s.
Cottage Cheese	**2**	[0 - 12]	**1**	[0 - 4]	**0**	[0 - 2]	**0**	[0 - 4]	**2**	[0 - 4]	**0.012***	n.s.
Cocoa	**0**	[0 - 2]	**0**	[0 - 3]	**0**	[0 - 2]	**0**	[0 - 2]	**0**	[0 - 2]	0.646	n.s.
Horlicks	**0**	[0 - 0]	**0**	[0 - 0]	**0**	[0 - 0]	**0**	[0 - 0]	**0**	[0 - 0]	**0.034***	n.s.
Coffee Whitener	**0**	[0 - 0]	**0**	[0 - 0]	**0**	[0 - 0]	**0**	[0 - 0]	**0**	[0 - 1]	0.144	n.s.

Eggs and egg products: Frequency of consumption (Median [P25-P75])

	**Pre**		**T1**		**T2**		**T3**		**Follow-up**		**FDR**	**Pairwise comparison**
Eggs	**4**	[2 - 12]	**4**	[2 - 6]	**2**	[2 - 4]	**2**	[0 - 4]	**4**	[4 - 12]	**< .001*****	**C, I, K**
Quiche	**0**	[0 - 2]	**0**	[0 - 2]	**0**	[0 - 2]	**0**	[0 - 2]	**0**	[0 - 2]	0.811	n.s.

Soups and Sauces: Frequency of consumption (Median [P25-P75])

	**Pre**		**T1**		**T2**		**T3**		**Follow-up**		**FDR**	**Pairwise comparison**
Low-cal Salad Cream	**0**	[0 - 0]	**0**	[0 - 0]	**0**	[0 - 0]	**0**	[0 - 0]	**0**	[0 - 0]	0.384	n.s.
Salad Cream	**0**	[0 - 2]	**0**	[0 - 2]	**0**	[0 - 2]	**0**	[0 - 0]	**2**	[0 - 2]	**0.002****	**K**
French Dressing	**0**	[0 - 12]	**0**	[0 - 4]	**0**	[0 - 2]	**0**	[0 - 0]	**0**	[0 - 2]	**< .001*****	n.s.
Vegetable Soup	**2**	[2 - 4]	**12**	[4 - 12]	**12**	[2 - 22]	**3**	[2 - 12]	**2**	[2 - 4]	**< .001*****	**A, B, G, I**
Meat Soup	**0**	[0 - 2]	**2**	[0 - 2]	**0**	[0 - 2]	**0**	[0 - 2]	**0**	[0 - 2]	0.182	n.s.
Sauces	**0**	[0 - 3]	**0**	[0 - 4]	**2**	[0 - 4]	**1**	[0 - 4]	**0**	[0 - 2]	0.973	n.s.
Ketchup	**0**	[0 - 2]	**0**	[0 - 2]	**0**	[0 - 3]	**2**	[0 - 4]	**0**	[0 - 2]	0.060	n.s.
Pickles	**0**	[0 - 2]	**0**	[0 - 2]	**0**	[0 - 3]	**0**	[0 - 2]	**2**	[0 - 2]	0.631	n.s.
Marmite	**0**	[0 - 0]	**0**	[0 - 0]	**0**	[0 - 0]	**0**	[0 - 0]	**0**	[0 - 0]	0.861	n.s.

Fats and Oils: Frequency of consumption (Median [P25-P75])

	Pre	T1	T2	T3	Follow-up	FDR	Pairwise comparison
Butter	**4** [2 - 15]	**2** [0 - 12]	**2** [0 - 12]	**0** [0 - 4]	**3** [0 - 4]	**0.008****	**C**
Hard Margarine	**0** [0 - 0]	**0** [0 - 0]	**0** [0 - 0]	**0** [0 - 0]	**0** [0 - 0]	0.779	n.s.
Polyunsaturated Margarine	**0** [0 - 0]	**0** [0 - 0]	**0** [0 - 0]	**0** [0 - 0]	**0** [0 - 0]	0.158	n.s.
Other Margarine	**0** [0 - 0]	**0** [0 - 0]	**0** [0 - 0]	**0** [0 - 0]	**0** [0 - 0]	0.501	n.s.
Low-fat Spread	**0** [0 - 0]	**0** [0 - 0]	**0** [0 - 0]	**0** [0 - 0]	**0** [0 - 0]	0.081	n.s.
Very Low-fat Spread	**0** [0 - 0]	**0** [0 - 0]	**0** [0 - 0]	**0** [0 - 0]	**0** [0 - 0]	0.345	n.s.

Sugars, Preserves and Snacks: Frequency of consumption (Median [P25-P75])

	Pre	T1	T2	T3	Follow-up	FDR	Pairwise comparison
Ice Cream	**2** [2 - 4]	**2** [0 - 2]	**2** [0 - 2]	**2** [0 - 4]	**2** [2 - 4]	**0.011***	n.s.
Chocolates	**4** [2 - 12]	**12** [2 - 22]	**12** [2 - 22]	**8** [2 - 22]	**4** [2 - 12]	0.074	n.s.
Chocolate Bars	**1** [0 - 2]	**2** [0 - 3]	**0** [0 - 2]	**0** [0 - 2]	**0** [0 - 0]	**0.008****	**G**
Sweets	**0** [0 - 2]	**0** [0 - 2]	**0** [0 - 12]	**0** [0 - 2]	**0** [0 - 0.5]	0.051	n.s.
Sugar	**8** [0 - 39]	**1** [0 - 24]	**0** [0 - 28]	**0** [0 - 28]	**2** [0 - 12]	0.129	n.s.
Crisps	**0** [0 - 2]	**0** [0 - 2]	**0** [0 - 0]	**0** [0 - 0]	**0** [0 - 2]	0.129	n.s.
Jam	**2** [2 - 15]	**2** [0 - 6]	**0** [0 - 3]	**0** [0 - 4]	**2** [0 - 4]	**< .001*****	**B, C**

Nuts and Seeds: Frequency of consumption (Median [P25-P75])

	Pre	T1	T2	T3	Follow-up	FDR	Pairwise comparison
Nuts	**2** [2 - 12]	**4** [2 - 12]	**4** [4 - 15]	**4** [2 - 12]	**4** [2 - 4]	**0.005****	**I**
Peanut Butter	**0** [0 - 0]	**0** [0 - 0]	**0** [0 - 0]	**0** [0 - 0]	**0** [0 - 2]	0.051	n.s.

Nonalcoholic Beverages: Frequency of consumption (Median [P25-P75])

	Pre	T1	T2	T3	Follow-up	FDR	Pairwise comparison
Tea	**12** [2 - 28]	**17** [2 - 39]	**22** [2 - 39]	**17** [2 - 28]	**8** [2 - 22]	0.185	n.s.
Instant Coffee	**28** [0 - 70]	**28** [0 - 70]	**25** [0 - 70]	**22** [0 - 70]	**22** [0 - 70]	**0.041***	n.s.
Decaff Coffee	**0** [0 - 1]	**0** [0 - 0]	**0** [0 - 0]	**0** [0 - 0]	**0** [0 - 1]	**0.003****	n.s.
Lowcal Fizzy Drinks	**0** [0 - 6]	**0** [0 - 4]	**0** [0 - 2]	**0** [0 - 2]	**0** [0 - 2]	0.122	n.s.
Fizzy Drinks	**0** [0 - 2]	**0** [0 - 4]	**0** [0 - 4]	**0** [0 - 4]	**0** [0 - 2]	0.715	n.s.
Fruit Juice	**4** [2 - 22]	**22** [4 - 28]	**12** [2 - 28]	**12** [2 - 24]	**2** [2 - 4]	**< .001*****	**G, I, K**

Alcoholic Beverages: Frequency of consumption (Median [P25-P75])

	Pre	T1	T2	T3	Follow-up	FDR	Pairwise comparison
Wine	**4** [2 - 12]	**8** [2 - 12]	**4** [2 - 12]	**4** [2 - 12]	**4** [2 - 12]	0.815	n.s.
Beer	**4** [2 - 12]	**12** [2 - 12]	**4** [0 - 12]	**4** [2 - 12]	**4** [4 - 12]	0.769	n.s.
Port	**0** [0 - 1]	**0** [0 - 4]	**0** [0 - 4]	**0** [0 - 4]	**0** [0 - 2]	0.142	n.s.
Spirits	**0** [0 - 2]	**0** [0 - 2]	**2** [0 - 4]	**0** [0 - 3]	**1** [0 - 2]	**0.020***	n.s.

Fruits: Frequency of consumption (Median [P25-P75])

	Pre	T1	T2	T3	Follow-up	FDR	Pairwise comparison
Apples	**4** [2 - 12]	**4** [2 - 15]	**2** [0 - 12]	**0** [0 - 4]	**4** [2 - 12]	**0.002****	**C, F, K**
Pears	**2** [2 - 6]	**0** [0 - 2]	**0** [0 - 0]	**0** [0 - 0]	**2** [0 - 4]	**< .001*****	**A, B, C, I, K**

Oranges	**12**	[2 - 12]	**4**	[2 - 12]	**0**	[0 - 0]	**0**	[0 - 0]	**4**	[2 - 12]	**< .001*** **	**B, C, E, F, I, K**
Grapefruit	**0**	[0 - 2]	**0**	[0 - 4]	**0**	[0 - 0]	**0**	[0 - 0]	**0**	[0 - 0]	**0.001** **	n.s.
Bananas	**4**	[2 - 12]	**2**	[0 - 4]	**0**	[0 - 0]	**0**	[0 - 0]	**4**	[2 - 12]	**< .001*** **	**B, C, F, I, K**
Grapes	**2**	[0 - 12]	**0**	[0 - 2]	**0**	[0 - 0]	**0**	[0 - 0]	**2**	[0 - 2]	**< .001*** **	**B, C, I, K**
Melons	**4**	[2 - 12]	**0**	[0 - 1]	**0**	[0 - 0]	**0**	[0 - 0]	**2**	[0 - 2]	**< .001*** **	**A, B, C, I, K**
Peaches	**2**	[0 - 12]	**0**	[0 - 0]	**0**	[0 - 0]	**0**	[0 - 0]	**2**	[0 - 4]	**< .001*** **	**A, B, C, I, K**
Strawberries	**4**	[2 - 12]	**1**	[0 - 4]	**0**	[0 - 0]	**0**	[0 - 0]	**4**	[2 - 12]	**< .001*** **	**B, C, G, I, K**
Tinned Fruit	**0**	[0 - 0]	**2**	[0 - 2]	**0**	[0 - 3]	**0**	[0 - 6]	**0**	[0 - 0]	**< .001*** **	**G**
Dried Fruit	**2**	[0 - 2]	**0**	[0 - 2]	**0**	[0 - 2]	**0**	[0 - 2]	**2**	[0 - 4]	**< .001*** **	**G, I, K**
Avocado	**2**	[0 - 3]	**0**	[0 - 2]	**0**	[0 - 0]	**0**	[0 - 0]	**2**	[0 - 2]	**< .001*** **	**B, C, F, I, K**

Vegetables: Frequency of consumption (Median [P25-P75])

	Pre		T1		T2		T3		Follow-up		FDR	Pairwise comparison
Carrots	**4**	[2 - 12]	**4**	[4 - 12]	**4**	[2 - 12]	**3**	[2 - 4]	**4**	[4 - 12]	0.159	n.s.
Spinach	**2**	[2 - 4]	**2**	[0 - 4]	**4**	[2 - 4]	**4**	[2 - 4]	**2**	[2 - 4]	0.121	n.s.
Broccoli	**4**	[2 - 4]	**4**	[2 - 12]	**4**	[0 - 4]	**4**	[0 - 4]	**2**	[2 - 4]	0.725	n.s.
Sprouts	**0**	[0 - 2]	**0**	[0 - 2]	**0**	[0 - 0]	**0**	[0 - 2]	**0**	[0 - 2]	0.375	n.s.
Cabbage	**2**	[0 - 4]	**2**	[0 - 3]	**0**	[0 - 2]	**0**	[0 - 0]	**2**	[2 - 4]	**< .001*** **	**C, F, K**
Peas	**2**	[2 - 4]	**2**	[2 - 4]	**2**	[2 - 4]	**2**	[0 - 4]	**2**	[2 - 4]	0.946	n.s.
Green Beans	**4**	[2 - 4]	**2**	[2 - 4]	**4**	[2 - 4]	**4**	[2 - 4]	**4**	[2 - 4]	0.523	n.s.
Marrow	**4**	[2 - 6]	**4**	[2 - 12]	**0**	[0 - 4]	**0**	[0 - 2]	**4**	[2 - 4]	**< .001*** **	**B, C, E, F, I, K**
Cauliflower	**2**	[0 - 4]	**2**	[0 - 3]	**0**	[0 - 4]	**0**	[0 - 2]	**2**	[2 - 4]	**< .001*** **	**C, K**
Parsnips	**0**	[0 - 0]	**0**	[0 - 0]	**0**	[0 - 0]	**0**	[0 - 0]	**0**	[0 - 2]	**< .001*** **	n.s.
Leeks	**2**	[0 - 4]	**0**	[0 - 0]	**0**	[0 - 0]	**0**	[0 - 0]	**2**	[0 - 4]	**< .001*** **	**A, C, G, I, K**
Onions	**8**	[2 - 12]	**4**	[2 - 12]	**4**	[2 - 12]	**0**	[0 - 2]	**12**	[4 - 12]	**< .001*** **	**C, F, H, K**
Garlic	**4**	[2 - 12]	**4**	[2 - 12]	**2**	[0 - 12]	**0**	[0 - 4]	**4**	[2 - 4]	**< .001*** **	**C, K**
Mushrooms	**2**	[2 - 12]	**2**	[2 - 4]	**2**	[2 - 4]	**2**	[0 - 4]	**4**	[2 - 4]	**0.023***	n.s.
Peppers	**2**	[2 - 6]	**2**	[0 - 4]	**2**	[2 - 4]	**1**	[0 - 4]	**2**	[2 - 4]	**0.013***	n.s.
Beansprouts	**0**	[0 - 0]	**0**	[0 - 0]	**0**	[0 - 0]	**0**	[0 - 0]	**0**	[0 - 0]	0.051	n.s.
Green Salad	**12**	[10 - 22]	**4**	[2 - 12]	**0**	[0 - 0]	**0**	[0 - 0]	**12**	[4 - 22]	**< .001*** **	**B, C, E, F, I, K**
Watercress	**0**	[0 - 0]	**0**	[0 - 0]	**0**	[0 - 0]	**0**	[0 - 0]	**0**	[0 - 0]	**0.022***	n.s.
Tomatoes	**12**	[10 - 22]	**4**	[4 - 12]	**0**	[0 - 0]	**0**	[0 - 0]	**12**	[4 - 12]	**< .001*** **	**B, C, E, F, I, K**
Sweetcorn	**2**	[0 - 4]	**0**	[0 - 4]	**0**	[0 - 2]	**0**	[0 - 0]	**2**	[0 - 2]	**< .001*** **	**C, K**
Beetroot	**2**	[0 - 2]	**0**	[0 - 3]	**0**	[0 - 1]	**0**	[0 - 0]	**0**	[0 - 2]	**< .001*** **	**C, F**
Coleslaw	**0**	[0 - 2]	**0**	[0 - 2]	**0**	[0 - 0]	**0**	[0 - 0]	**0**	[0 - 2]	**< .001*** **	**C**
Beans	**0**	[0 - 0]	**0**	[0 - 0]	**0**	[0 - 0]	**0**	[0 - 0]	**0**	[0 - 0]	0.739	n.s.
Tofu	**0**	[0 - 1]	**0**	[0 - 0]	**0**	[0 - 0]	**0**	[0 - 0]	**0**	[0 - 2]	**< .001*** **	n.s.

Notes: Consumption frequencies per month are presented as median [P25–P75] for each time point: before (Pre), during (T1 = February, T2 = May, T3 = October), and six months after (Follow-up) the Antarctic mission. Statistical analysis was conducted using the Friedman test, followed by Bonferroni-adjusted post hoc comparisons. *P*-values were corrected for multiple testing using the false discovery rate (FDR). Significance levels: *FDR = .01–.05; **FDR = .001–.01; ***FDR < .001. Pairwise comparisons from the Bonferroni-adjusted post hoc test are denoted by letters: A = Pre-T1, B = Pre-T2, C = Pre-T3, D = Pre-Follow-up, E = T1-T2, F = T1-T3, G = T1- Follow-up, H = T2-T3, I = T2- Follow-up, K = T3-Follow-up. *Abbreviations:* n.s. = not significant.

Supplementary Table S4: Energy-adjusted micronutrient density expressed per 100 kcal of total intake.

	Pre	T1	T2	T3	Follow-up	FDR	Pairwise comparisons
**Major minerals**							
Calcium [mg/100 kcal]	40.72 ± 12.21	37.28 ± 10.72	34.72 ± 8.9	37.11 ± 15.48	49.49 ± 16.13	< 0.001***	B, G, I, K
Chloride [mg/100 kcal]	175.4 ± 33.5	188.06 ± 34.76	195.19 ± 40.06	183.1 ± 46.96	187.31 ± 33.37	0.236	n.s.
Magnesium [mg/100 kcal]	16.64 ± 3.31	15.94 ± 2.84	15.32 ± 2.69	15.22 ± 2.78	17.17 ± 3.37	0.02*	I, K
Phosphorus [mg/100 kcal]	72.85 ± 9.46	70.46 ± 12.08	68.14 ± 10.32	72.5 ± 15	77.78 ± 13.18	0.012*	I
Potassium [mg/100 kcal]	183.35 ± 35.47	171.12 ± 35.72	157.13 ± 22.59	155.06 ± 37.02	181.46 ± 30.87	< 0.001***	B, I
Sodium [mg/100 kcal]	118.78 ± 22.38	127.94 ± 22.97	133.12 ± 26.43	126.59 ± 29.94	125.78 ± 22.04	0.304	n.s.
**Trace Elements**							
Copper [mg/100 kcal]	0.07 ± 0.02	0.06 ± 0.01	0.06 ± 0.01	0.06 ± 0.02	0.07 ± 0.02	0.537	n.s.
Iodine [mcg/100 kcal]	7.51 ± 2.59	7.56 ± 2.82	6.93 ± 1.88	7.68 ± 3.51	8.16 ± 2.46	0.304	n.s.
Iron [mg/100 kcal]	0.59 ± 0.12	0.6 ± 0.09	0.58 ± 0.09	0.6 ± 0.11	0.6 ± 0.11	0.758	n.s.
Manganese [mg/100 kcal]	0.17 ± 0.07	0.16 ± 0.04	0.16 ± 0.05	0.15 ± 0.05	0.17 ± 0.07	0.260	n.s.
Selenium [mcg/100 kcal]	4.03 ± 1.07	4.11 ± 1.22	3.82 ± 1.02	4.32 ± 1.09	4.04 ± 1.22	0.216	n.s.
Zinc [mg/100 kcal]	0.55 ± 0.12	0.55 ± 0.11	0.54 ± 0.13	0.59 ± 0.21	0.56 ± 0.11	0.381	n.s.
**Vitamins fat-soluble**							
Vitamin A [mcg/100 kcal]	25.43 ± 30.99	23.94 ± 24.02	19.15 ± 16.75	21.54 ± 36.21	26.09 ± 28.93	0.023*	C, K
Vitamin A equivalents [mcg/100 kcal]	58.96 ± 36.75	52.22 ± 30.44	43.66 ± 18.74	48.28 ± 40.27	58.47 ± 35.6	0.023*	C, K
Vitamin D [mcg/100 kcal]	0.16 ± 0.08	0.16 ± 0.07	0.14 ± 0.07	0.15 ± 0.07	0.16 ± 0.07	0.635	n.s.
Vitamin E [mg/100 kcal]	0.56 ± 0.15	0.54 ± 0.15	0.58 ± 0.19	0.48 ± 0.15	0.55 ± 0.14	0.053	n.s.
**Vitamins water-soluble**							
Vitamin B1 [mg/100 kcal]	0.07 ± 0.01	0.07 ± 0.01	0.07 ± 0.01	0.07 ± 0.02	0.07 ± 0.01	0.444	n.s.
Vitamin B2 [mg/100 kcal]	0.09 ± 0.02	0.09 ± 0.03	0.08 ± 0.04	0.08 ± 0.04	0.09 ± 0.03	0.304	n.s.
Vitamin B6 [mg/100 kcal]	0.11 ± 0.02	0.11 ± 0.02	0.1 ± 0.03	0.11 ± 0.04	0.11 ± 0.02	0.697	n.s.
Vitamin B12 [mcg/100 kcal]	0.36 ± 0.17	0.39 ± 0.17	0.32 ± 0.14	0.39 ± 0.16	0.37 ± 0.16	0.279	n.s.
Vitamin C [mg/100 kcal]	6.54 ± 2.76	5.82 ± 3.36	4.04 ± 2.27	3.55 ± 1.94	5.41 ± 1.91	< 0.001***	B, C, K
Niacin [mg/100 kcal]	1.31 ± 0.39	1.33 ± 0.31	1.36 ± 0.28	1.46 ± 0.5	1.24 ± 0.35	0.236	n.s.
Folate (total) [mcg/100 kcal]	13.93 ± 3.68	13.81 ± 4.68	13.24 ± 4.16	12.84 ± 4.36	13.99 ± 3.45	0.459	n.s.

Notes: Daily intake of minerals [mg/mcg] and vitamins [mg/mcg] per 100 kcal of total intake presented as mean ± SD. Statistical significance was assessed using repeated measures analysis with false discovery rate (FDR) correction. Significance levels: *FDR = .01–.05; **FDR = .001–.01; ***FDR < .001. Pairwise comparisons from the Bonferroni-adjusted post hoc test are denoted by letters: A = Pre–T1, B = Pre–T2, C = Pre–T3, D = Pre– Follow-up, E = T1–T2, F = T1–T3, G = T1– Follow-up, H = T2–T3, I = T2– Follow-up, K = T3–Follow-up. *Abbreviations:* n.s. = not significant.
